# Prognostic value of the CIPA nutritional screening tool in over 30,000 hospitalized patients: a retrospective study (2014–2022)

**DOI:** 10.3389/fnut.2025.1648228

**Published:** 2025-09-24

**Authors:** Javier García Fernández, José Pablo Suárez-Llanos, Cristina Lorenzo González, Elena Márquez Mesa, María Araceli García Núñez, Maria Demelza Farrais Luis, Manuel Enrique Fuentes Ferrer

**Affiliations:** ^1^Department of Endocrinology and Nutrition, Nuestra Señora de Candelaria University Hospital, Santa Cruz de Tenerife, Spain; ^2^Department of Preventive Medicine, Nuestra Señora de Candelaria University Hospital, Santa Cruz de Tenerife, Spain

**Keywords:** nutrition assessment, malnutrition, mortality, length of stay, readmission, prognosis

## Abstract

**Introduction:**

Malnutrition is a well-established negative prognostic factor in hospitalized patients, contributing to increased morbidity and mortality. The CIPA (Control of Food Intake, Protein, and Anthropometry) screening tool was developed to identify patients at nutritional risk and to predict adverse clinical outcomes across both surgical and non-surgical populations. This study aimed to evaluate the prognostic value of the CIPA tool in routine clinical practice by analyzing its association with key clinical outcomes since its implementation at our center in 2014.

**Materials and methods:**

We conducted a retrospective analysis of inpatients screened with the CIPA tool between 2014 and 2022 in a tertiary care hospital. The association between CIPA screening results and clinical outcomes—including 3- and 6-month mortality, early hospital readmission, and length of stay—was assessed, with a particular focus on patients with active oncological disease. Regression analyses were adjusted for age, sex, admitting department, and type of admission.

**Results:**

A total of 30,581 patients were included, of whom 31.4% screened positive for malnutrition using the CIPA tool. CIPA-positive patients had significantly higher mortality at 3 months (adjusted OR 3.02; 95% CI: 2.78–3.30; *p* < 0.001) and at 6 months (adjusted OR 2.69; 95% CI: 2.49–2.91; *p* < 0.001). They also exhibited increased rates of early readmission (adjusted OR 1.43; 95% CI: 1.34–1.53; *p* < 0.001) and a longer median hospital stay (*β* = 0.25; 95% CI: 0.23–0.27; *p* < 0.001).

**Discussion:**

In this large, real-world cohort, the CIPA nutritional screening tool was a robust predictor of poorer clinical outcomes among hospitalized patients with positive screening results. These findings support the utility of CIPA screening for early identification of high-risk patients, enabling targeted nutritional interventions to potentially mitigate adverse outcomes.

## 1 Introduction

Disease-associated malnutrition is a significant prognostic factor in hospitalized patients, correlating with higher rates of complications, diminished treatment efficacy, and impaired immune responses. These adverse effects contribute to prolonged hospital stays, increased early readmission rates, elevated healthcare expenditures, and higher mortality (1). The prevalence of malnutrition in hospitalized populations remains alarmingly high, with reported rates ranging from 23 to 33%, as evidenced by large-scale multicenter studies conducted in Spain, including PREDyCES (2) and seDREno (3), as well as the EUROOPS (4) study encompassing countries in Europe and North Africa.

Malnutrition is also highly prevalent among oncology patients (10–50%), driven by both tumor-related factors and the impact of treatment. It adversely affects treatment efficacy, increases toxicity, and compromises both survival and quality of life (3, 5). Moreover, it is estimated to contribute to mortality in 10–20% of cancer patients (6). Nonetheless, according to a nationwide survey conducted in the United States in 2019, just over 50% of cancer centers performed nutritional screening, and only 35% of those utilized validated methods (7).

Several studies have demonstrated that nutritional intervention can improve clinical outcomes in malnourished patients, including those with cancer (8), and can be cost-effective. These findings underscore the importance of early diagnosis of malnutrition upon admission and throughout hospitalization (6). However, malnutrition often remains underdiagnosed, partly because universal nutritional screening is not routinely implemented in hospitals—despite several studies suggesting that such programs are cost-effective (9, 10).

In 2019, the Global Leadership Initiative on Malnutrition (GLIM), comprising representatives from leading Clinical Nutrition societies, established global consensus criteria for diagnosing disease-related malnutrition (11). This method involves two steps: first, a screening test to identify malnourished patients or those at risk, followed by a diagnostic assessment of malnutrition and its severity. However, no standardized, globally implemented nutritional screening tool exists to perform this initial step. In 2025, GLIM updated its consensus criteria, refining the diagnostic approach and incorporating new evidence (12).

An ideal tool should be tailored to the hospital’s characteristics, be practical and simple, and effectively identify patients at risk of poor clinical outcomes based on nutritional parameters (13). At Nuestra Señora de Candelaria University Hospital (HUNSC), a nutritional screening tool called CIPA (Control of Intakes, Proteins, and Anthropometry) has been developed, validated, and implemented since 2014, meeting these criteria and adopted by other hospitals as well (14, 15). With extensive experience using CIPA, we have conducted this retrospective observational study analyzing over 30,000 screenings to evaluate the clinical outcomes of patients based on screening results, with a particular focus on oncological patients.

## 2 Materials and methods

### 2.1 Study type

This was a retrospective observational study based on a clinical cohort of hospitalized patients between 2014 and 2022 at Nuestra Señora de Candelaria University Hospital (HUNSC), in whom nutritional screening using the CIPA tool was performed upon admission.

### 2.2 Setting

HUNSC is a tertiary care hospital located on the island of Tenerife. It is the largest hospital in the autonomous community of the Canary Islands, Spain, and serves a catchment population of over 500,000 people.

The study was approved by the HUNSC Research Ethics Committee (project code: CHUNSC_2023_55). It was conducted in accordance with the principles outlined in the Declaration of Helsinki (Fortaleza revision, Brazil, October 2013) and current European and Spanish regulations.

### 2.3 Inclusion and exclusion criteria

Eligible participants were patients aged 16 years or older who were admitted to hospital services and underwent CIPA screening between January 1, 2014, and December 31, 2022. Patients were included if CIPA screening could be defined based on the available data. Specifically, patients were classified as CIPA positive if at least one of the three CIPA components — oral intake during the first 72 h of admission, plasma albumin level on admission, or body mass index (BMI) / mid-upper arm circumference (MUAC) on admission — was available in the hospital’s electronic health record (EHR) and positive. In contrast, to be classified as CIPA negative, information on all three components had to be available and all had to be negative. Exclusion criteria comprised an expected hospital stay of less than 72 h; admission to obstetrics, pediatrics, palliative care, or critical care units; and departments with a low prevalence of malnutrition (e.g., ophthalmology, dermatology). Patients receiving nutritional therapy (parenteral, enteral, or oral nutritional supplements) at the time of screening were also excluded.

### 2.4 Data collection and analysis

All patients underwent the CIPA nutritional screening routinely used at our hospital. Screening was conducted on day 3 of hospitalization, and results were recorded in the EHR. For the present study, the variables required for the CIPA screening were obtained from the EHR through automated queries. The CIPA screening is implemented in the EHR as a standardized form, developed specifically for this purpose and systematically completed by nursing staff. This form records the three CIPA components: (a) oral intake during the first 72 h of admission, documented daily by nursing staff; (b) plasma albumin on admission, automatically determined in the first blood test performed at admission; and (c) BMI or, when not available, MUAC measured on the first day of hospitalization. While albumin and anthropometric measures are available from day 1, the final CIPA result is defined on day 3, once 72-h food intake has been assessed. A positive result in any of these components constituted a positive CIPA screening (14). All CIPA-related data were entered into the EHR by nursing staff.

In addition to CIPA screening results, demographic and clinical variables (age, sex, admitting department, type of admission, and presence of active oncological disease) were also retrieved from the EHR in collaboration with the Hospital Management Service. The dataset was pseudonymized before being exported into a secure research database for analysis. Diagnostic codes were extracted using the 10th revision of the International Classification of Diseases (ICD-10) and subsequently grouped into broader diagnostic categories.

When the screening was positive, the attending physician was notified and nutritional support was initiated according to hospital protocols, which could include dietary counseling, oral nutritional supplements, or enteral/parenteral nutrition depending on the patient’s condition. In routine clinical practice, if the initial screening is negative, CIPA is repeated every 10 days during hospitalization; however, for the present study only the first screening performed at day 3 was considered.

Mortality at 3 and 6 months was obtained through the EHR, which is linked to the regional database and routinely updated with mortality information. For each patient, deaths occurring within 3 or 6 months of the date of the index hospitalization were identified, and mortality at these time points was calculated accordingly. Hospital readmissions were retrieved using the same system, which prospectively records all admissions and discharges within the regional network. Each hospital admission was considered as a new patient record. In cases where the same individual had multiple admissions during the study period (2014–2022), hospitalizations corresponding to the same episode of care (i.e., readmissions directly related to the index hospitalization) were consolidated and not treated as separate records. This approach ensured consistency in the calculation of outcome variables and avoided double-counting patients with multiple admissions.

### 2.5 Data statistical analysis

Qualitative variables were summarized as frequency distribution, and normally distributed quantitative variables as mean ± standard deviation (SD). The continuous, non-normally distributed variables were summarized as median and interquartile range (IQR). To assess the skewness of quantitative variables, a graphical inspection of histograms and box plots, together with quantile-quantile normality plots, was performed.

Comparison of variables according to the CIPA nutritional screening tool result (+/−) was performed using the chi-square test for qualitative variables and the Student’s *t*-test for quantitative variables, or the nonparametric Mann–Whitney U test if applicable.

The relationship of the outcome variables (3 and 6 month mortality and readmission for 30 days) with the CIPA nutritional screening tool result was assessed using binary logistic regression. For the outcome variable length of stay, a linear regression model was fitted. As the length of stay was not normally distributed, this data was log-transformed. Each model was adjusted by age, sex, admission department, type of admission, and active oncological disease.

To assess whether the effect of the CIPA nutritional screening result on each outcome variable varied according to the presence of active oncological disease, an interaction term between CIPA and active oncological disease was included in the previously specified models.

Statistical significance was assumed as *p* < 0.05. All analyses were performed using SPSS 26.0 (IBM Corp., Armonk, NY, USA).

## 3 Results

### 3.1 Descriptive analysis

A total of 30,581 CIPA nutritional screenings were collected on patients admitted to our center between 2014 and 2022. There was a slight predominance of males (55.5%), with an average patient age of 65.2 ± 16.3 years. The highest percentage of screenings was performed in patients aged 65 to 74 years (24.2%), while the lowest occurred in those under 35 years (4.9%).

A positive CIPA nutritional screening result was observed in 9,614 patients (31.4%). Among these, 15.3% reported decreased intake, 6.5% had a BMI below 20 kg/m^2^, and 16.5% had serum albumin levels under 3 g/dL.

The majority of patients analyzed were admitted urgently (27,744; 90.7%) compared to those with scheduled admissions. Mortality rates at 3 and 6 months following hospital admission were 9.0% (2,767 patients) and 11.7% (3,570 patients), respectively. The 30-day readmission rate was 16.3%. The median hospital length of stay was 10 days (IQR: 6–17).

**Figure 1:**
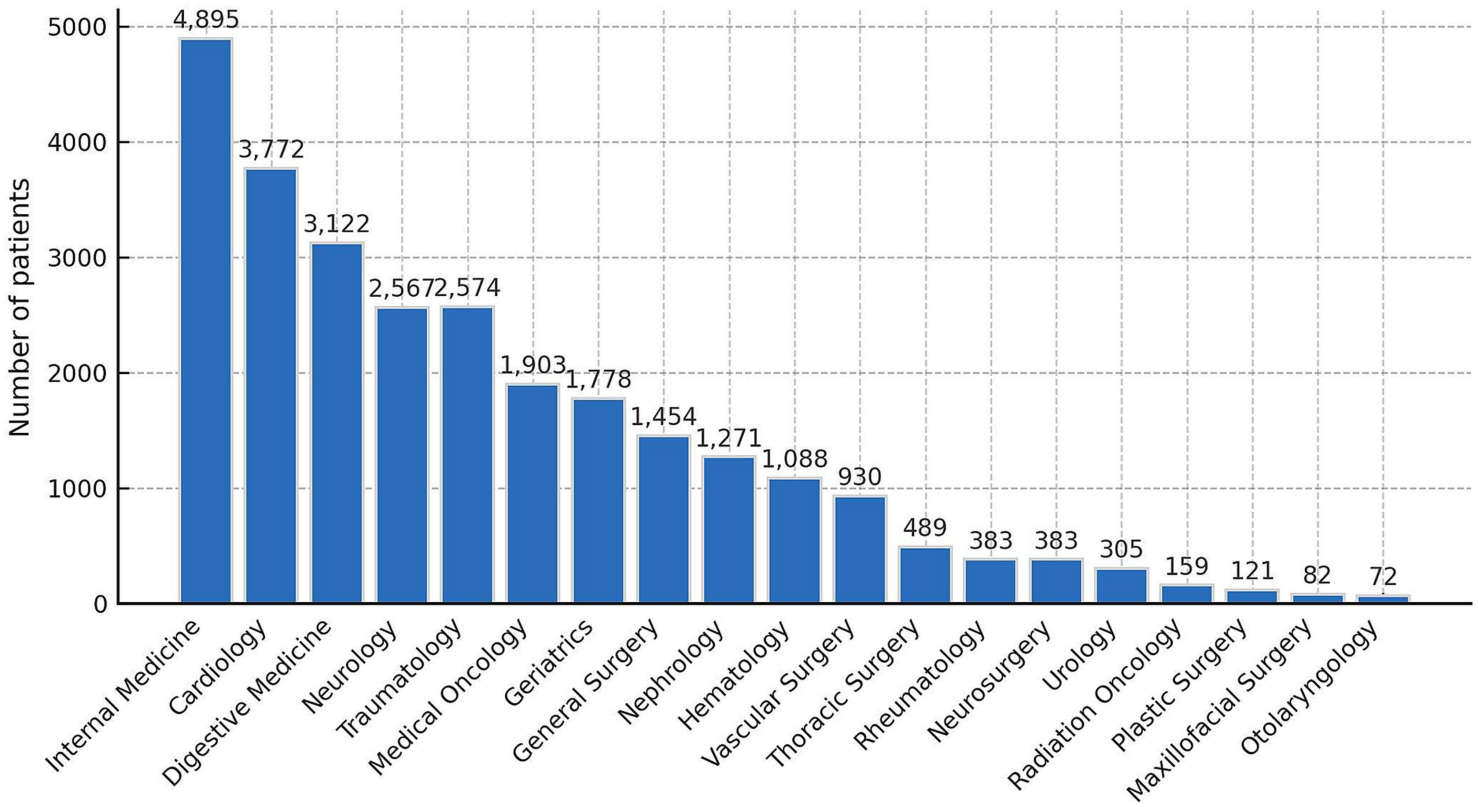
Overall patients distribution by admission department for patients undergoing malnutrition screening CIPA.

In terms of hospital service distribution, 79% of nutritional screenings were conducted in medical departments, compared to 21% in surgical departments. The highest number of screenings occurred in Internal Medicine (4,895; 16.0%), Cardiology (3,772; 12.3%), Pneumology (3,233; 10.6%), and Digestive (3,122; 10.2%). Departments with the lowest number of screenings were Otorhinolaryngology (72; 0.2%), Maxillofacial Surgery (82; 0.3%), and Plastic Surgery (121; 0.4%). Within surgical services, Traumatology accounted for the most screenings of the total sample (2,574; 8.4%). A complete distribution by admission department is shown in Figure 1.

The primary diagnoses leading to hospital admission were analyzed using the 10th edition of the International Classification of Diseases (ICD-10). The most frequent diagnoses were diseases of the circulatory system (7,651; 25%), followed by diseases of the respiratory system (4,381; 14.3%) and digestive system (4,013; 13.1%). A total of 3,247 patients (10.6%) had active oncological disease (Table 1).

**Table 1:** Primary diagnoses leading to hospital admission among patients who underwent CIPA nutritional screening classified using the 10th edition of the International Classification of Diseases (ICD-10).

Primary diagnosis	*n* (%)
Diseases of the circulatory system	7,651 (25%)
Diseases of the respiratory system	4,381 (14.3%)
Diseases of the digestive system	4,013 (13.1%)
Neoplasms	3,247 (10.6%)
Diseases of the genitourinary system	2,789 (9.1%)
Diseases of the musculoskeletal system and connective tissue	2,456 (8%)
Diseases of the nervous system	1,975 (6.5%)
Endocrine, nutritional and metabolic diseases	1,863 (6.1%)
Infectious and parasitic diseases	1,489 (4.9%)
Injury, poisoning and certain other consequences of external causes	716 (2.3%)
Mental and behavioral disorders	2 (0.1%)

### 3.2 Prevalence of malnutrition according to CIPA screening result and its association with outcome variables

**Figure 2:**
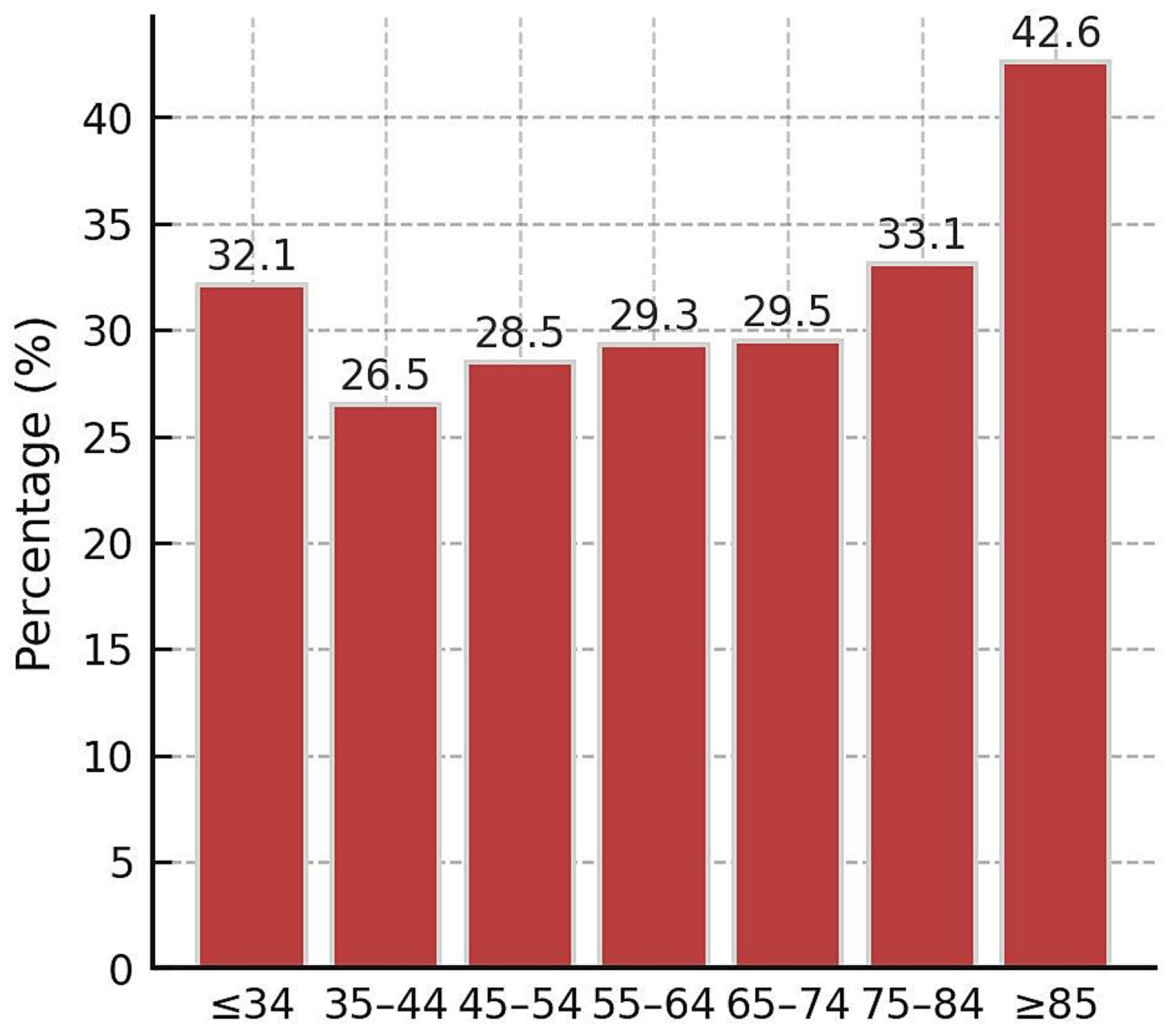
Frequency of positive CIPA nutritional screening by age groups.

Statistically significant differences were found when analyzing the positive CIPA result by age, sex, admission department (medical or surgical), and active oncological disease. The prevalence of malnutrition was highest among women (34.9%) compared to men (28.6%), in surgical departments (36.6%) versus medical services (30.1%), and in patients with active oncological disease (39%) compared to those without (29.4%), However, no relationship was found between the positive screening result and the type of admission (urgent or scheduled) (Table 2). The frequency of malnutrition also appears to increase with the age of patients with positive CIPA (Figure 2).

**Table 2:** Frequency of CIPA nutritional screening in relation to clinical and sociodemographic characteristics.

	Total n	CIPA-Positive n (%)	CIPA-Negative n (%)	*p*
Age, mean (±SD)	65.2 (± 16.3)	66.7 (**±**16.8)	64.6 (**±**16)	<0.001

Gender				
Males	16,959	4,854 (28.6)	12,105 (71.4)	<0.001
Females	13,622	4,760 (34.9)	8,862 (65.1)
AD				
Medical	24,171	7,267 (30.1)	16,904 (69.9)	<0.001
Surgical	6,410	2,347 (36.6)	4,063 (63.4)	
TA				
Urgent	27,744	8,768 (31.6)	18,976 (68.4)	0.051
Scheduled	2,837	846 (29.8)	1,991 (70.2)	
AOD				
Yes	6,457	2,516 (39.0)	3,941 (61.0)		<0.001
No	24,124	7,098 (29.4)	17,026 (70.6)		

AD, Admission department; TA, Type of admission; AOD, Active oncological disease; SD, standard deviation.

**Figure 3:**
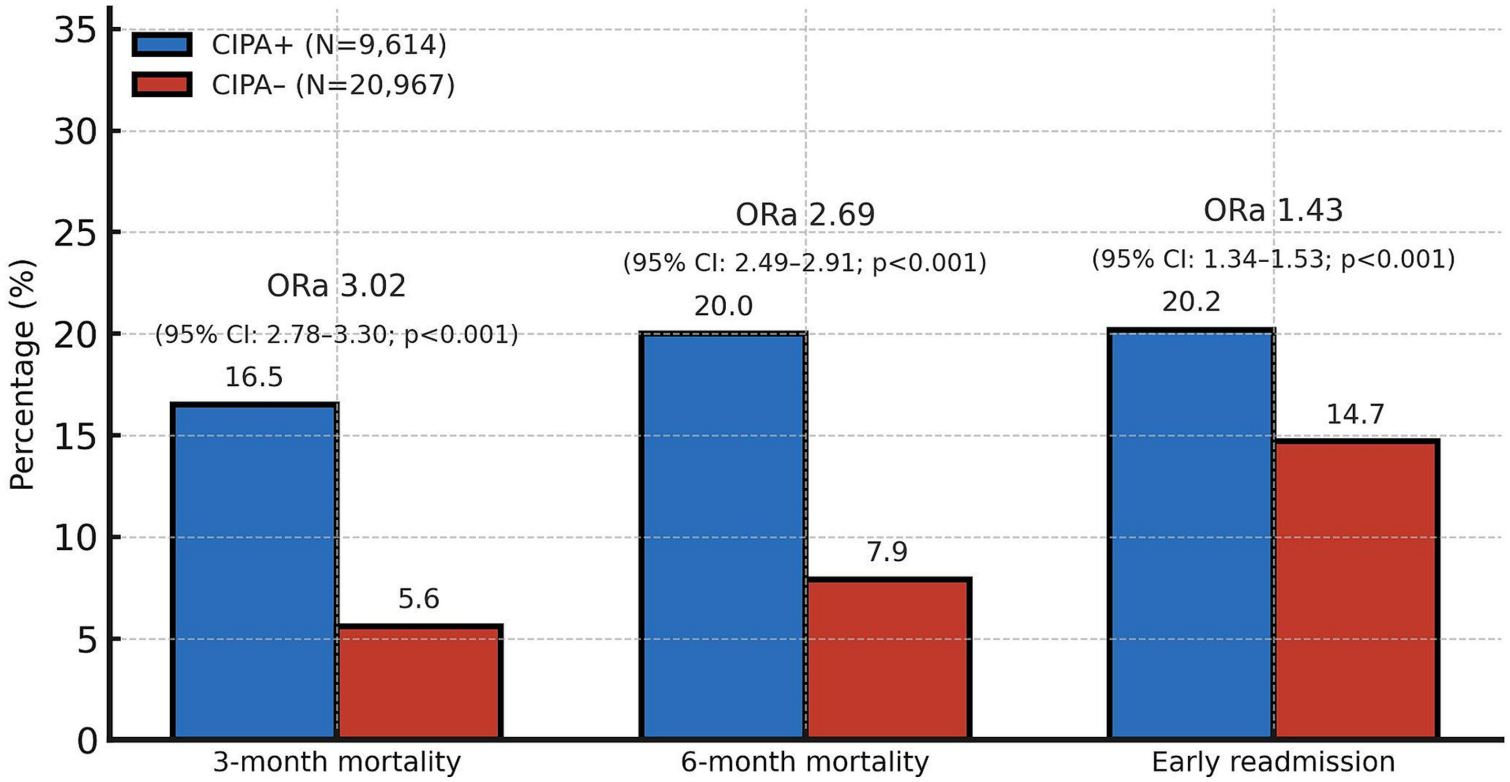
Association of CIPA nutritional screening results with early readmission and 3-month and 6-month mortality. ORa, adjusted odds ratio; CI, confidence interval. The adjusted odds ratios represent the association between CIPA-positive and CIPA-negative patients for each binary outcome (3- and 6-month mortality and early readmission). ORa values were obtained from multivariate binary logistic regression analyses, adjusted for age, sex, admission department, type of admission, and active oncological disease, using a binary logistic regression model.

Three- and six-month mortality rates, as well as early readmission rates, were significantly (*p* < 0,001) higher among patients with a positive CIPA nutritional screening (16.5, 20.0, and 20.2%, respectively) compared to those with a negative screening (5.6, 7.9, and 14.7%, respectively). After adjusting for age, sex, admission department, type of admission, and active oncological disease using a binary logistic regression model, these associations remained significant: adjusted odds ratios (ORa) for three-month mortality, six-month mortality, and early readmission were 3.02 (95% CI: 2.78–3.30; *p* < 0.001), 2.69 (95% CI: 2.49–2.91; *p* < 0.001), and 1.43 (95% CI: 1.34–1.53; *p* < 0.001), respectively (Figure 3).

The median length of stay was statistically higher in the group with a positive CIPA nutritional screening result (median: 12 days; IQR: 8–22) than in the group with a negative result (median: 9 days, IQR: 6–16; *p* < 0.001). In the adjusted linear regression analysis the average length of hospital stay was significantly higher among patients with a positive screening result (adjusted *β*: 0.25; 95% CI: 0.23–0.27; *p* < 0.001).

**Figure 4:**
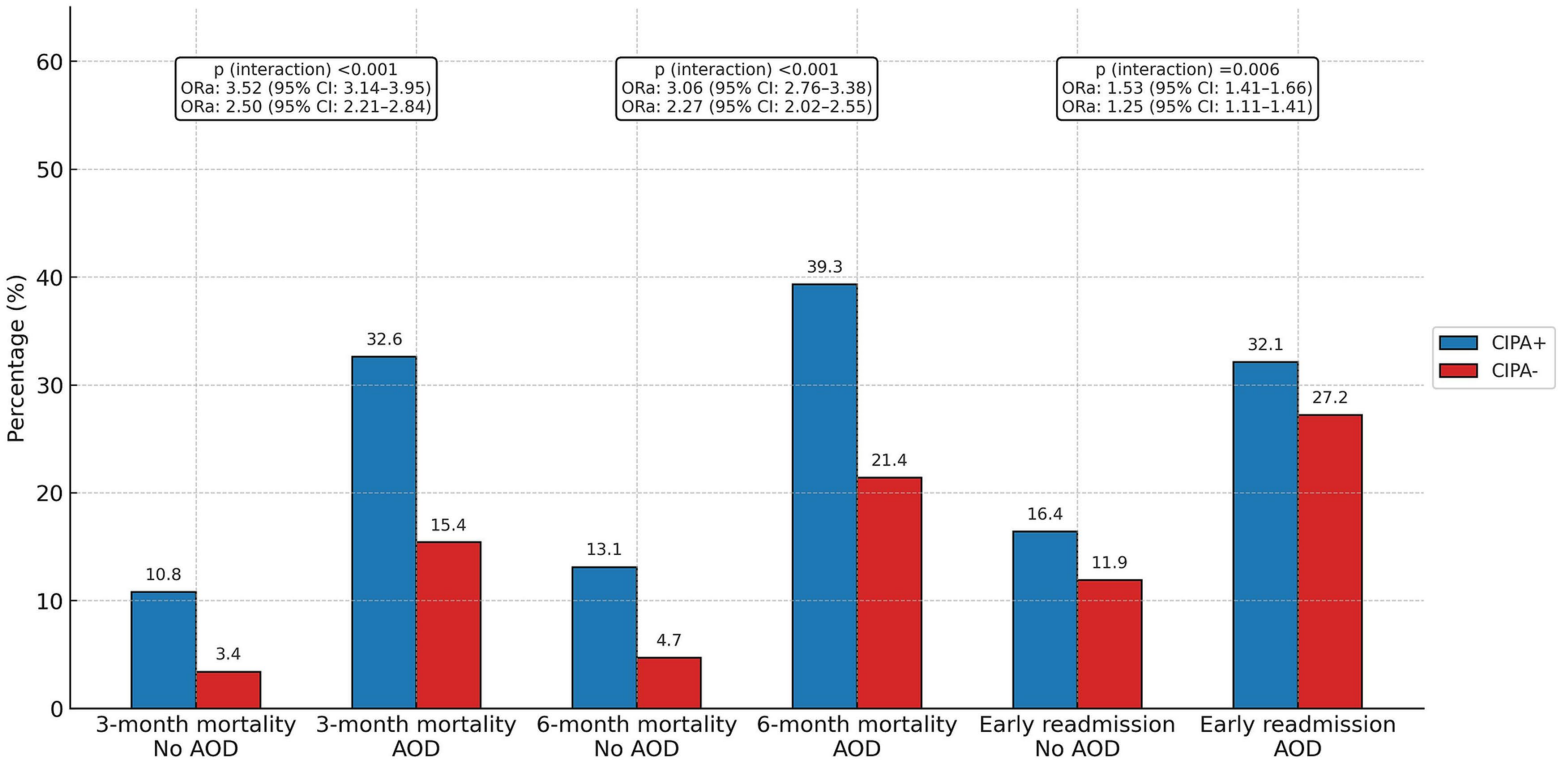
Association of CIPA nutritional screening results with early readmission and 3- and 6-month mortality according to the diagnosis of active oncological disease. AOD, active oncological disease; ORa, adjusted odds ratio; CI, confidence interval. The adjusted odds ratios represent the association between patients with positive and negative CIPA results for each binary outcome variable (3- and 6-month mortality and early readmission), stratified by the diagnosis of active oncological disease. ORa values were obtained from multivariate binary logistic regression analyses adjusted for age, sex, admission department, type of admission, and active oncological disease, using a binary logistic regression model. Interaction *p*-values indicate the statistical significance of the interaction term introduced in the multivariate logistic regression to evaluate whether the effect of the CIPA nutritional screening result on each outcome variable varied according to the presence of active oncological disease.

In the stratified analysis, multivariate analyses adjusted for age, sex, admission department and type of admission were conducted to evaluate three- and six-month mortality and early readmission rates based on the presence or absence of active oncological disease. In both the group of patients with active oncological disease and the group without it, significant associations were found between a positive CIPA screening result and the outcomes of mortality at 3 and 6 months, as well as 30-day readmission (Figure 4). Among patients with oncological disease, these associations were weaker for both 3- and 6-month mortality (*p* < 0.001) and 30-day readmission (*p* = 0.006) compared to those observed in patients without this condition.

Among patients with active oncological disease, those with a positive CIPA nutritional screening had a significantly longer median hospital stay (12 days, IQR 7–20) than those with a negative screening (9 days, IQR 6–17; *p* < 0.001). A similar trend was observed in patients without active oncological disease: the median stay was 13 days (IQR 8–22) for CIPA-positive cases versus 9 days (IQR 6–15) for CIPA-negative cases (*p* < 0.001). Considering these findings, stratified analysis indicated that the impact of a positive CIPA result in increasing the length of hospital stay was statistically (p < 0.001) more pronounced in the non-active oncological disease group (adjusted *β*: 0.28; 95% CI: 0.26–0.30; *p* < 0.001) compared to the active oncological disease group (adjusted *β* 0.13; 95% CI: 0.10–0.17; *p* < 0.001).

## 4 Discussion

Disease-related malnutrition remains a major healthcare challenge, with clear clinical and economic consequences, including longer hospital stays, higher readmission rates, and increased mortality (1). In our study, about one-third of hospitalized patients screened positive with the CIPA tool, and these patients showed significantly worse outcomes in terms of 3- and 6-month mortality, 30-day readmission, and hospital stay.

In terms of baseline characteristics, the sample had a mean age of 65 years, with a balanced gender distribution. Regarding service distribution, Internal Medicine, Cardiology, Gastroenterology, and Neurology represented nearly half of the total sample. These baseline characteristics align with those of similar studies, such as the previously mentioned seDREno (3), where the sample had a mean age of 67 years and 65.7% were admitted to a medical service. This alignment is consistent with the typical patient profile in tertiary hospitals and is a notable strength of this study, as it reflects the diversity of patients admitted to these centers rather than focusing on a specific pathology.

Our study found a malnutrition prevalence of 31.4%, a rate somewhat lower than previously reported figures using the same CIPA malnutrition screening tool [36.5% (16), 35.8% (10), and 35.4% (15)]. These discrepancies may be partly explained by differences in inclusion criteria and case mix: the cost-effectiveness study (10) focused only on Internal Medicine and Digestive Surgery patients; the surgical inpatient study (15) included exclusively surgical cases; and the cross-sectional study using body composition analysis (16) likely selected patients with more complex comorbidity profiles.

In contrast to these prior findings, our observed prevalence aligns more closely with that of the SeDREno study (3), which reported a prevalence of 29.7% using MUST and GLIM criteria. Importantly, SeDREno applied inclusion and exclusion criteria very similar to ours (e.g., excluding short stays, ICU, pediatrics, obstetrics, psychiatry, dermatology, ophthalmology, and palliative care), which makes the close agreement in prevalence between both studies particularly consistent. Interestingly, the PREDyCES study (2) also used highly similar inclusion and exclusion criteria, yet reported a substantially lower prevalence of 23% when applying the NRS-2002 screening tool. However, in the EuroOOPS study (4), which also employed the NRS-2002 across 26 hospitals in Europe and the Middle East, the prevalence of nutritional risk was 32.6%, almost identical to our findings.

Taken together, these comparisons suggest that methodological differences in the screening tools used largely explain the variability in prevalence figures across studies. Age distribution may also play a role: the mean age in our cohort (65.2 ± 16.3 years) was higher than in EuroOOPS (59.8 ± 0.3 years) (4), yet comparable to SeDREno (67.1 ± 17 years) (3) and PREDyCES (mean ranging from 60.5 ± 17.4 years in controls to 73.7 ± 12.6 years in patients at nutritional risk) (2). Notably, malnutrition prevalence among subgroups aged >70 years increased to 34.8 and 37% in PREDyCES and SeDREno, respectively, which is in line with the prevalence observed in our work. These consistencies reinforce the robustness and external validity of our findings. Furthermore, the recent study by da Silva et al. reported malnutrition prevalence as high as 61–63% when utilizing GLIM criteria in older populations (17).

Given this context, the well-documented clinical, economic, and quality-of-life benefits of nutritional interventions for hospital malnutrition further underscore the need for universal nutritional screening within hospital settings (18). Despite initial evidence suggesting cost-effectiveness (10), implementing such screening remains challenging due to the increased workload on healthcare personnel and the resource commitment required by institutions—factors that have met with resistance from some administrators.

With respect to the key clinical outcomes, patients with a positive CIPA screening had higher mortality at 3 and 6 months. The sub-analysis of the EFFORT study conducted in Switzerland also found that the NRS-2002 nutritional screening was able to significantly predict higher mortality in patients with a positive result (19).

Concerning the early readmission rate, it was also significantly higher in patients with a positive CIPA screening, completing the ominous clinical triangle of these patients. Major studies like seDREno (3) also manage to detect patients at higher risk of readmission through other nutritional screenings such as MUST or NRS2002.

Regarding the last clinical objective, such as median hospital stay, the fact that patients with a positive CIPA screening had a longer hospital stay likely reflects the higher incidence of hospital complications that malnourished patients may present, including infectious, post-surgical, or lower functional capacity. But it also implies a higher healthcare cost that may be relevant when convincing managers and politicians of the importance of implementing universal nutritional screening in all hospitals. In most studies conducted with the CIPA screening, positive patients stayed in the hospital for one more week than negative ones; the greater heterogeneity of this sample and the lack of active intervention may be the causes of this difference, without diminishing the relevance of the results found in this work. With other nutritional screening tools such as the MUST used in the seDREno study, NRS 2002 used in the EuroOOPS, or SGA (20), this correlation between positive screening and increased hospital stay has also been observed.

Notably, if analyzing all outcome variables collectively, the associations of CIPA screening positive result with three- and six-month mortality were qualitatively approximately twice as strong as those observed for early readmission rates.

Interestingly, notable differences were observed in prognostic outcomes compared to a recent study (16) that assessed CIPA and GLIM criteria for the diagnosis of malnutrition. In the referenced study, neither patients with positive CIPA screening results nor those meeting GLIM criteria exhibited a significant increase in hospital readmission rates. The reasons underlying this discrepancy with our findings remain unclear. It is important to note that our subgroup analysis revealed a significantly higher readmission rate among patients with active oncological disease. Nonetheless, it appears logical that patients identified by any screening tool as being at high risk for malnutrition or classified as malnourished would exhibit significantly higher hospital readmission rates compared to well-nourished individuals, consistent with the results of this study.

Malnutrition is also particularly common in cancer patients and is again associated with adverse outcomes, especially in the elderly population (21). In fact, recently published American clinical practice guidelines recommend that all oncology patients, not only those hospitalized, undergo nutritional screening using a validated tool following a cancer diagnosis and subsequently throughout treatment (22). Similarly, earlier prominent European reviews had also advocated for nutritional screening in oncology patients (23). In line with this, our study demonstrates that patients undergoing nutritional screening who are identified as malnourished or at risk of malnutrition experience worse outcomes in terms of mortality and length of stay. These findings are also consistent with those of a systematic review and meta-analysis (5), as well as with those of Liu et al. (24), published recently using GLIM criteria.

The CIPA nutritional screening, one of the first implemented in Spain, offered a unique opportunity to analyze a large cohort of patients assessed within the context of routine hospital clinical practice, providing insights into a ‘real-life’ setting. We consider this to be one of the main strengths of the study. Moreover, the extensive sample size enhanced the statistical power, ensuring robust and reliable results. Finally, while this study was retrospective, it allowed for the evaluation of patient prognosis without the control biases often introduced in prospective studies. These strengths make our study comparable to other large-scale analyses of malnutrition risk.

Our findings are consistent with previous large-scale studies that evaluated malnutrition risk in hospitalized patients. Carrera-Gil et al. (25), in a unicentric cohort of 11,722 admissions, showed that nutritional risk assessed with the MST was independently associated with higher in-hospital mortality (OR = 2.32) and ICU admission (OR = 1.13). Likewise, Meulemans et al. (26), in a multicenter propensity score–matched cohort of 73,843 patients, found that NRS-2002–positive patients had higher in-hospital mortality (OR = 1.56), 30-day mortality (OR = 1.62), and readmission within 4 months (OR = 1.12). As previously shown in our study, CIPA-positive patients also had higher 3-month mortality (OR = 3.02), 6-month mortality (OR = 2.69), and early readmission (OR = 1.43), These effect sizes are therefore highly consistent across different tools and populations, reinforcing the robustness of malnutrition risk as a prognostic marker. However, our study specifically addresses the CIPA tool, providing the first large-scale evidence of its applicability and prognostic value when implemented in routine hospital practice, thereby complementing the evidence generated with other screening instruments.

This study has several limitations, primarily related to its retrospective design and reliance on routinely collected EHR data. Relevant prognostic variables, including comorbidities, were often missing or inconsistently recorded, which limited adequate risk adjustment and may have introduced residual confounding. The study population was also heterogeneous, encompassing patients with diverse clinical profiles and conditions, which further complicated the retrieval of detailed prognostic information.

Another limitation of our study is the potential for selection bias, since patients without sufficient information to calculate the CIPA score could not be included. However, as previously discussed, the prevalence observed in our cohort was similar to that reported in other studies conducted both in our center and across Europe, suggesting that our estimates are robust and unlikely to be substantially biased.

Moreover, we were unable to determine the proportion of CIPA-positive patients who received nutritional intervention, due to limitations in the electronic prescription system used to request nutritional support. This represents an important limitation, as the observed associations between CIPA positivity and adverse outcomes may have been attenuated by the beneficial effect of nutritional treatment. Nonetheless, as had been robustly demonstrated (27), nutritional intervention in malnourished or at-risk patients, improves outcomes, suggesting that the differences observed in our study might have been even more pronounced if no patients had received nutritional support.

We also acknowledge that, although only patients with at least one positive CIPA component were included, we did not perform a detailed analysis of the independent prognostic value of each component, since the study was designed to evaluate the tool as a whole. Future studies could explore the relative contribution of oral intake, albumin, and BMI/MUAC to the overall prognostic performance of CIPA.

Finally, the external validity of our findings should be interpreted with caution. Since this study was conducted in a single tertiary hospital, differences in patient case mix, staffing resources, and organizational practices may limit the direct generalizability of results to other healthcare contexts. However, the CIPA tool is the nutritional screening method recommended by the Canary Islands Health Service and has already been implemented in four other hospitals across the region. This regional adoption suggests that our findings may be applicable to a wider range of hospital settings and supports the feasibility of integrating CIPA into routine clinical practice beyond our center.

In summary, our study further demonstrates the prognostic value of the CIPA nutritional screening tool in a highly heterogeneous and representative hospital population. Despite the limitations of its retrospective design, the consistency of our findings with large national and European cohorts supports their validity. Mortality at 3 and 6 months, readmission rates, and length of stay were significantly higher in CIPA-positive patients, including those with active oncological disease. These results emphasize the need for standardized hospital-wide nutritional screening to identify at-risk patients and implement individualized nutritional plans aimed at preventing complications and improving patient prognosis.

## Data Availability

The raw data supporting the conclusions of this article will be made available by the authors, without undue reservation.

## References

[1] Ruiz AJ Buitrago G Rodríguez N Gómez G Sulo S Gómez C. Clinical and economic outcomes associated with malnutrition in hospitalized patients. Clin Nutr. (2019) 38:1310–6. doi: 10.1016/j.clnu.2018.05.016, PMID: 29891224

[2] León-Sanz M Brosa M Planas M García-de-Lorenzo A Celaya-Pérez S Hernández JÁ. Predyces study: the cost of hospital malnutrition in Spain. Nutrition. (2015) 31:1096–102. doi: 10.1016/j.nut.2015.03.009, PMID: 26233866

[3] Zugasti-Murillo A Petrina-Jáuregui ME Ripa-Ciáurriz C Sánchez-Sánchez R Villazón-González F Faes ÁGD. SeDREno study — prevalence of hospital malnutrition according to glim criteria, ten years after the PREDyCES study. Nutr Hosp. (2021) 38:1016–25. doi: 10.20960/nh.03638 34157845

[4] Sorensen J Kondrup J Prokopowicz J Schiesser M Krähenbühl L Meier R. EuroOOPS: an international, multicentre study to implement nutritional risk screening and evaluate clinical outcome. Clin Nutr. (2008) 27:340–9. doi: 10.1016/j.clnu.2008.03.012, PMID: 18504063

[5] Peng D Zong K Yang H Huang Z Mou T Jiang P. Malnutrition diagnosed by the global leadership initiative on malnutrition criteria predicting survival and clinical outcomes of patients with cancer: a systematic review and meta-analysis. Front Nutr. (2022) 9:1053165. doi: 10.3389/fnut.2022.1053165, PMID: 36562033 PMC9763567

[6] Muscaritoli M Arends J Bachmann P Baracos V Barthelemy N Bertz H. ESPEN practical guideline: Clinical Nutrition in cancer. Clin Nutr. (2021) 40:2898–913. doi: 10.1016/j.clnu.2021.02.005, PMID: 33946039

[7] Trujillo EB Claghorn K Dixon SW Hill EB Braun A Lipinski E. Inadequate nutrition coverage in outpatient cancer centers: results of a national survey. J Oncol. (2019) 2019:7462940. doi: 10.1155/2019/7462940 31885583 PMC6893237

[8] Virizuela JA Camblor-Álvarez M Luengo-Pérez LM Grande E Álvarez-Hernández J Sendrós-Madroño MJ. Nutritional support and parenteral nutrition in cancer patients: an expert consensus report. Clin Transl Oncol. (2018) 20:619–29. doi: 10.1007/s12094-017-1757-4, PMID: 29043569

[9] Kruizenga HM Van Tulder MW Seidell JC Thijs A Ader HJ. Effectiveness and cost-effectiveness of early screening and treatment of malnourished patients. Am J Clin Nutr. (2005) 82:1082–9. doi: 10.1093/ajcn/82.5.1082, PMID: 16280442

[10] Suárez-Llanos JP Vallejo-Torres L García-Bello MÁ Hernández-Carballo C Calderón-Ledezma EM Rosat-Rodrigo A. Cost-effectiveness of the hospital nutrition screening tool CIPA. Arch Med Sci. (2020) 16:273–81. doi: 10.5114/aoms.2018.81128, PMID: 32190136 PMC7069439

[11] Cederholm T Jensen GL Correia MITD Gonzalez MC Fukushima R Higashiguchi T. GLIM criteria for the diagnosis of malnutrition – a consensus report from the global clinical nutrition community. Clin Nutr. (2019) 38:1–9. doi: 10.1016/j.clnu.2018.08.002, PMID: 30181091

[12] Cederholm T Jensen GL Correia MITD Gonzalez MC Fukushima R Pisprasert V. The GLIM consensus approach to diagnosis of malnutrition: a 5-year update. Clin Nutr. (2025) 49:11–20. doi: 10.1016/j.clnu.2025.03.018, PMID: 40222089

[13] Ferguson M Capra S Bauer J Banks M. Development of a valid and reliable malnutrition screening tool for adult acute hospital patients. Nutrition. (1999) 15:458–64. doi: 10.1016/S0899-9007(99)00084-2, PMID: 10378201

[14] Suárez-Llanos JP Mora-Mendoza A Benítez-Brito N Pérez-Méndez L Pereyra-García-Castro F Oliva-García JG. Validity of the new nutrition screening tool control of food intake, protein, and anthropometry (CIPA) in non-surgical inpatients. Arch Med Sci. (2018) 14:1020–4. doi: 10.5114/aoms.2017.66084, PMID: 30154883 PMC6111349

[15] Mendoza AM Llanos JPS Morales AS González CL Huerta YZ de Segura ILG. Validation of CIPA nutritional screening through prognostic clinical variables in hospitalized surgical patients. Endocrinol Diabetes Nutr. (2020) 67:304–9. doi: 10.1016/j.endinu.2019.07.008, PMID: 31668927

[16] Márquez Mesa E Guerra Cabrera AJ Gómez de Segura IL Suárez Llanos JP. Comparison of CIPA nutritional screening with GLIM criteria for malnutrition, prognostic evolution, and association with phase angle in hospitalized patients. Nutrients. (2024) 16:3652. doi: 10.3390/nu16213652, PMID: 39519485 PMC11547199

[17] da Silva GD Batista AVDA Costa MCDA Dos Santos AC. The ability of GLIM and MNA-FF to diagnose malnutrition and predict sarcopenia and frailty in hospitalized adults over 60 years of age. Front Nutr. (2024) 11:1456091. doi: 10.3389/fnut.2024.1456091 39582663 PMC11583805

[18] House M Gwaltney C. Malnutrition screening and diagnosis tools: implications for practice. Nutr Clin Pract. (2022) 37:12–22. doi: 10.1002/ncp.10801, PMID: 34897800

[19] Hersberger L Bargetzi L Bargetzi A Tribolet P Fehr R Baechli V. Nutritional risk screening (NRS 2002) is a strong and modifiable predictor risk score for short-term and long-term clinical outcomes: secondary analysis of a prospective randomised trial. Clin Nutr. (2020) 39:2720–9. doi: 10.1016/j.clnu.2019.11.041, PMID: 31882232

[20] Aparecida Leandro-Merhi V Braga De Aquino JL Sales Chagas JF. Nutrition status and risk factors associated with length of hospital stay for surgical patients. JPEN J Parenter Enteral Nutr. (2011) 35:241–8. doi: 10.1177/0148607110374477, PMID: 20971940

[21] Zhang Q Qian L Liu T Ding JS Zhang X Song MM. Prevalence and prognostic value of malnutrition among elderly Cancer patients using three scoring systems. Front Nutr. (2021) 8:738550. doi: 10.3389/fnut.2021.738550, PMID: 34708064 PMC8544751

[22] Trujillo EB Kadakia KC Thomson C Zhang FF Livinski A Pollard K. Malnutrition risk screening in adult oncology outpatients: an ASPEN systematic review and clinical recommendations. J Parenter Enter Nutr. (2024) 48:874–94. doi: 10.1002/jpen.2688, PMID: 39412097

[23] Aprile G Basile D Giaretta R Schiavo G La Verde N Corradi E. The clinical value of nutritional care before and during active Cancer treatment. Nutrients. (2021) 13:1196. doi: 10.3390/nu13041196, PMID: 33916385 PMC8065908

[24] Liu C Lu Z Li Z Xu J Cui H Zhu M. Influence of malnutrition according to the GLIM criteria on the clinical outcomes of hospitalized patients with cancer. Front Nutr. (2021) 24:774636. doi: 10.3389/fnut.2021.774636 PMC8739964 35004809

[25] Carrera-Gil F Prieto Rusca MI. Efficiency of a technology-assisted nutritional screening system: a retrospective analysis of 11,722 admissions in a tertiary hospital. Clin Nutr ESPEN. (2024) 64:51–6. doi: 10.1016/j.clnesp.2024.08.022, PMID: 39214246

[26] Meulemans A Matthys C Vangoitsenhoven R Sabino J Van Der Schueren B Maertens P. A multicenter propensity score matched analysis in 73,843 patients of an association of nutritional risk with mortality, length of stay and readmission rates. Am J Clin Nutr. (2021) 114:1123–30. doi: 10.1093/ajcn/nqab135, PMID: 33987635

[27] Schuetz P Fehr R Baechli V Geiser M Deiss M Gomes F. Individualised nutritional support in medical inpatients at nutritional risk: a randomised clinical trial. Lancet (London, England). (2019) 393:2312–21. doi: 10.1016/S0140-6736(18)32776-4, PMID: 31030981

